# LPC-DHA/EPA-Enriched Diets Increase Brain DHA and Modulate Behavior in Mice That Express Human *APOE4*

**DOI:** 10.3389/fnins.2021.690410

**Published:** 2021-07-01

**Authors:** Sarah B. Scheinman, Dhavamani Sugasini, Monay Zayed, Poorna C. R. Yalagala, Felecia M. Marottoli, Papasani V. Subbaiah, Leon M. Tai

**Affiliations:** ^1^Department of Anatomy and Cell Biology, University of Illinois at Chicago, Chicago, IL, United States; ^2^Division of Endocrinology and Metabolism, Department of Medicine, University of Illinois at Chicago, Chicago, IL, United States; ^3^Jesse Brown VA Medical Center, Chicago, IL, United States

**Keywords:** DHA, LPC-DHA, *APOE4*, memory, aging

## Abstract

Compared with *APOE3*, *APOE4* is associated with greater age-related cognitive decline and higher risk of neurodegenerative disorders. Therefore, development of supplements that target *APOE* genotype-modulated processes could provide a great benefit for the aging population. Evidence suggests a link between *APOE* genotype and docosahexaenoic acid (DHA); however, clinical studies with current DHA supplements have produced negative results in dementia. The lack of beneficial effects with current DHA supplements may be related to limited bioavailability, as the optimal form of DHA for brain uptake is lysophosphatidylcholine (LPC)-DHA. We previously developed a method to enrich the LPC-DHA content of krill oil through lipase treatment (LT-krill oil), which resulted in fivefold higher enrichment in brain DHA levels in wild-type mice compared with untreated krill oil. Here, we evaluated the effect of a control diet, diet containing krill oil, or a diet containing LT-krill oil in *APOE3*- and *APOE4-*targeted replacement mice (*APOE*-TR mice; treated from 4 to 12 months of age). We found that DHA levels in the plasma and hippocampus are lower in *APOE4*-TR mice and that LT-krill oil increased DHA levels in the plasma and hippocampus of both *APOE3*- and *APOE4*-TR mice. In *APOE4*-TR mice, LT-krill oil treatment resulted in higher levels of the synaptic vesicle protein SV2A and improved performance on the novel object recognition test. In conclusion, our data demonstrate that LPC-DHA/EPA-enriched krill oil can increase brain DHA and improve memory-relevant behavior in mice that express *APOE4*. Therefore, long-term use of LT-krill oil supplements may on some level protect against age-related neurodegeneration.

## Introduction

The human *APOE* genotype is linked to several adult-onset neurodegenerative disorders, as *APOE4* is associated with greater age-related cognitive decline, poorer outcomes following brain trauma, and higher risk of developing Alzheimer’s disease (Liu et al., 2013; Mahley, 2016; Flowers and Rebeck, 2020) compared with *APOE3*. Therefore, identifying supplements that mitigate *APOE4-*associated behavioral deficits could provide a great benefit for delaying the onset of neurodegenerative disorders. As an apolipoprotein (apo), apoE contributes to lipid homeostasis in the periphery and the brain, which is considered particularly important for docosahexaenoic acid (DHA) (Abdullah et al., 2017; Nock et al., 2017; Patrick, 2019). DHA is an essential fatty acid involved in multiple processes that maintain neuron function, and there are reports of altered DHA levels in Alzheimer’s disease patients (Cunnane et al., 2013; Lo Van et al., 2016). *APOE* is connected to DHA in two important ways. The first is the general concept that *APOE4* predisposes neurons to dysfunction during aging, and therefore, *APOE4* carriers may require additional DHA for reparative processes (Patrick, 2019). The second is more direct, as *APOE4* may lower the amount of dietary DHA delivered to cells in the brain (Hennebelle et al., 2014; Nock et al., 2017; Patrick, 2019). Therefore, with *APOE4*, the combination of a greater DHA requirement to maintain neuronal function, and lower brain DHA enrichment from dietary sources, could collectively contribute to cognitive decline during aging. DHA supplements could provide some level of protection against cognitive decline in *APOE4* carriers, yet clinical studies have produced negative results in Alzheimer’s disease patients (reviewed in Yassine et al., 2017a). The lack of activity for current supplements in *APOE4* carriers may be related to limited DHA bioavailability (Vandal et al., 2014; Arellanes et al., 2020; Tomaszewski et al., 2020), supporting the need to identify other supplements that can enrich brain DHA.

Evidence supports that the optimal form of DHA for brain uptake is lysophosphatidylcholine (LPC)-DHA (Sugasini et al., 2017, 2019), whereas DHA in most available supplements is absorbed in triacylglycerol form. For example, DHA is the form of triacylglycerol in fish/algal oil, phospholipid in krill oil, and free fatty acid and ethyl esters in formulations. These dietary DHA compounds are hydrolyzed to free DHA by pancreatic enzymes (lipase for triacylglycerol and ethyl esters, phospholipase A2 for phosphatidyl choline), which is absorbed by the intestinal mucosa, converted to triacylglycerol, and secreted in chylomicrons (Sugasini et al., 2017, 2019). In contrast, LPC-DHA is resistant to hydrolysis by lipase or phospholipase A2 and is therefore either absorbed and transported as LPC-DHA or converted to phosphatidylcholine DHA in the intestinal mucosa that can be converted to LPC-DHA. The major pathway for DHA uptake into the brain was identified as the LPC-DHA specific Na-symporter, Mfsd2a (Nguyen et al., 2014). We have previously demonstrated that dietary LPC-DHA markedly enriches brain DHA, whereas other molecular forms of dietary DHA are ineffective (Sugasini et al., 2017, 2019). Furthermore, we showed that pretreatment of dietary krill oil with lipase similarly enables enrichment of brain DHA because of the conversion of sn-2 DHA-PC of krill oil to LPC-DHA (Yalagala et al., 2020). Therefore, the goal of this study was to evaluate the activity of lipase-treated krill oil at enriching brain DHA levels and improving behavior in mice that express human *APOE4*. To this end, we treated female and male *APOE3*- and *APOE4-*targeted replacement mice with control diet, diet containing krill oil, or a diet containing lipase-treated krill oil and assessed plasma and brain DHA levels, memory-relevant behavior, and neuronal protein markers.

## Materials and Methods

### Diet Preparation and Dose Calculations

Diets were prepared as previously described in Yalagala et al. (2020). Briefly, 15 g of krill oil (a generous gift from Bioriginal Food and Science Corporation, Saskatoon, Canada) was dissolved in 300 ml of 95% ethanol and 20 g immobilized lipase from *Mucor meihei* was added (Creative Enzymes, Shirley, NY, United States). The reaction mixture was incubated at 40°C in the dark for 72 h (under nitrogen, orbital incubator at 175 rpm), immobilized lipase separated, ethanol evaporated (vacuum), and lipids were extracted. Lipid extracts of control and lipase-treated krill oil were analyzed by TLC using iodine vapors. Only lanes containing standards were exposed to iodine, and the silica gel from the sample lane corresponding to standard LPC, PC, or PE was scraped, eluted with chloroform:methanol:water (65:25:4 by vol), methylated, and analyzed by gas chromatography/mass spectroscopy (GC/MS).

Three diets were utilized in this study: control, krill oil, and lipase-treated krill oil. Lipase treatment of krill oil results in enrichment of both LPC-DHA and LPC-eicosapentaenoic acid (LPC-EPA). The amounts of lipase-treated krill oil were selected to match our previous studies, where 3.6 μmol of LPC (2.6 μmol LPC-EPA + 1.0 μmol LPC-DHA) per 3 g of diet (average daily diet consumption per mouse) was effective at enriching brain DHA levels (Yalagala et al., 2020). Final diets of krill oil and lipase-treated krill oil contained similar levels of total DHA and EPA. The total amount of EPA + DHA in the preparation (LPC^+^ non-LPC) was 203 mg per g oil (130 mg EPA + 70 mg DHA), and the final diets contained 13 g krill oil (lipase treated or untreated) and 57 g of corn oil per kg to give 7% total fat in the diet. The human equivalent dose of LPC-EPA/DHA, calculated using allometric scaling, is about 0.8 mmol per day per 70 kg body weight. All oils were blended with AIN93G rodent diet, pelleted, and vacuum sealed by Dyets Inc. (Bethlehem, PA, United States). The diets were stored at −20°C and were thawed weekly before use.

### Mouse Model and Treatment

Experiments were conducted with ethical approval from UIC Institutional Animal Care and Use Committee protocols (approval number 18-074). Female and male *APOE*-targeted replacement (*APOE*-TR, Taconic, Albany, NY, United States) mice were used in this study as they express the human *APOE3* or *APOE4* under the control of the endogenous murine *APOE* promoter. *APOE3*-TR and *APOE4*-TR mice were treated from 4 to 12 months of age with a diet containing either krill oil treated with lipase, untreated krill oil, or corn oil. Mice were housed five per cage and fed the diets *ad libitum*; the food was changed three times a week and total food consumption was determined by weighing the remaining food. Body weights of mice from all treatment conditions were recorded every 2 weeks. All behavioral testing was conducted blinded; however, due to issues related to COVID-19, biochemical analysis was conducted unblinded.

### Behavioral Testing

Behavioral testing was conducted as described in Thomas et al. (2016); Marottoli et al. (2017), Thomas et al. (2017), and Marottoli et al. (2019) at treatment endpoint. Analyses were conducted during the mouse dark cycle, tracked in real time by a camera, and the videos analyzed using ANY-maze software (Stoelting, Wood Dale, IL, United States).

#### Open Field

Mice were placed in the center of a white box (*l* 413 mm × *w* 305 mm × *h* 300 mm) that contained a thin layer of bedding for a duration of 7 min. Total distance traveled and total time spent immobile were measured (Thomas et al., 2016, 2017; Marottoli et al., 2017, 2019), and mice that had a total distance traveled of less than 10 m were excluded from analysis.

#### Novel Object Recognition

Twenty-four hours after the open-field test, mice were placed in a white box (*l* 413 mm × *w* 305 mm × *h* 300 mm) containing two identical objects for 7 min. Mice were returned to their home cage for 1 h, and then placed in the testing chamber for another 7 min with a familiar and a novel object. Preference index (ratio of time spent with the novel object divided by the total investigation time of both objects) was calculated (Thomas et al., 2016, 2017; Marottoli et al., 2017, 2019), and mice that had a total investigation time of less than 10 s for both objects were excluded from analysis.

### Tissue Processing

Following behavioral testing, mice were fasted overnight and anesthetized with 100 mg/kg ketamine and 10 mg/kg xylazine (i.p.). Blood was drawn by cardiac puncture, mice were transcardially perfused with ice-cold phosphate-buffered saline, and the brain was then further dissected into the hippocampus. All samples were flash frozen in liquid nitrogen and stored at −80°C until analysis.

### Lipid Extraction and Fatty Acid Analysis

Lipids were extracted from plasma and brain tissue as described in Sugasini et al. (2017). Briefly, tissue was homogenized at 4°C in a glass homogenizer (50% methanol, 0.01 N HCl) with internal standards, chloroform added, samples were vortexed and centrifuged, and lipids were concentrated under nitrogen and redissolved in chloroform. LC/MS analysis was utilized for quantification of LPC species using Atlantis (Waters Corp., Milford, MA, United States) HILIC column and by multiple reaction monitoring in positive mode electrospray mass spectrometry (Yalagala et al., 2020). Quantification of LPC species was performed with the internal standard of 17:0, and a correction factor of (× 0.233) was applied to account for the differences in ion intensity of 17:0 LPC and 22:6 LPC species. For fatty acid analysis, lipids were extracted by the Bligh and Dyer procedure, and fatty acid methyl esters were prepared with methanolic HCl and analyzed by GC/MS (Sugasini et al., 2017).

### Western Blot Analysis

Hippocampal samples were processed as described previously (Youmans et al., 2012; Tai et al., 2013, 2014; Thomas et al., 2016, 2017; Marottoli et al., 2017, 2019) with slight modifications. Tissue was homogenized using a bead mill (Fisherbrand, Waltham, MA, United States) at 6 m/s for 1 cycle of 30 s in lysis buffer (1% SDS + 10 mM NaF + 2 mM Na_3_VO_4_ + 1 × protease inhibitor cocktail in 20 mM HEPES; pH = 7.4), after which samples were centrifuged at 500 × *g* for 5 min 4°C, sonicated (20% amplification, 3 cycles), and then centrifuged again (100,000 × *g* for 20 min). Aliquots of the resulting supernatant were then flash frozen in liquid nitrogen and stored −80°C. Protein content of each sample was quantified using the Pierce BCA Protein Assay Kit.

SV2A (Abcam Ab32942), PSD95 (Cell Signaling 3409S), and synaptophysin (Cell Signaling 5461) levels in the hippocampus were measured by Western blot analysis (Thomas et al., 2016, 2017; Marottoli et al., 2017, 2019). Protein (20 μg) was separated on 4–12% Bis-Tris gels (Invitrogen, Carlsbad, CA, United States), transferred onto low fluorescence PVDF membranes, blocked for 1 h at room temperature with 5% milk in TBS, washed 3 × 5 min with 0.1% Tween-20 in TBS (TBS-T), and probed with primary antibody overnight at 4°C in 1% bovine serum albumin in TBS with 0.02% sodium azide. Immediately following washing (3 × 5 min, TBS-T), membranes were incubated for 45 min at room temperature in appropriate secondary fluorescent antibodies (LI-COR, Lincoln, NE, United States, 800CW Donkey α-Rabbit IgG; LI-COR, 680RD Goat α-Mouse IgG) in 1% milk in TBS-T and 0.01% SDS. All proteins were imaged and quantified using the Odyssey Fc Imaging System and normalized to GAPDH.

### Statistical Analysis

Data are presented as mean ± SEM and were analyzed using three-way ANOVA followed by Tukey’s multiple comparison test, or by using Student’s *t*-test with GraphPad Prism version 8.3.1. See Supplementary File 1 for *n* sizes and statistical comparisons.

## Results

The goal of this study was to evaluate the ability of an LPC-DHA-enriched diet to increase brain DHA levels and improve memory-relevant behavior in mice that express human *APOE4*. Therefore, we treated male and female *APOE3*-TR and *APOE4*-TR mice with diets containing lipase-treated krill oil (LT-krill oil), krill oil, or no additional DHA (control diet, corn oil) from 4 to 12 months of age. We selected *APOE*-TR mice as they are a well-characterized model of *APOE*-modulated changes in behavior (Bour et al., 2008; Rodriguez et al., 2013), and utilized a prevention paradigm to mimic longer-term supplement use. Previously, we developed a method to enrich the LPC-DHA content of krill oil through treatment with lipase (Yalagala et al., 2020), which also increases LPC-EPA levels that may be relevant for neuropsychiatric symptoms of dementia. We selected krill oil, as it is the most prevalent and available phospholipid form of omega 3 in the market that can generate LPC-EPA/DHA through lipase reaction. EPA and DHA are found almost exclusively at the sn-2 position of phospholipids in krill oil and, therefore, are concentrated in LPC forms after lipase treatment. The total fatty acid composition of krill oil before and after lipase reaction is the same. In the LPC fraction of LT-krill oil, the content of EPA is ∼41.7% and DHA is ∼35.4% of total fatty acids (Yalagala et al., 2020; Supplementary File 1). In LT-krill oil, all PC and PE is hydrolyzed to LPC and LPE; however, LPE levels are minimal (Yalagala et al., 2020; Supplementary File 1) as the amount of PE in krill oil is much lower compared with PC (83% PC, 14% PE; Xie et al., 2019). The percentage of EPA is more than DHA in LT-krill oil, and the bioavailability of EPA and of DHA are the same, since both are taken up by the same transporter (Mfsd2a). However, after treatment with LT-krill oil, DHA accumulates in the brain to a greater extent than EPA, since EPA is largely converted to DHA either before or after entering the brain (Yalagala et al., 2020). We have previously demonstrated that LT-krill oil enriches brain DHA and EPA levels to a greater extent than krill oil in wild-type mice (Yalagala et al., 2020), which formed the basis for the current study; total DHA and EPA concentrations in each diet were 0.091 and 0.169 g per 100 g diet, respectively. Body weights, food consumption, and behavior were measured in all mice (*n* = 16–23). Plasma and hippocampal lipid levels were measured in a subset of mice (*n* = 5–6) and synaptic proteins (*n* = 7) in a different group (see Supplementary File 1 for specific *n* sizes and statistical analysis tables). Data were analyzed by three-way ANOVA (independent variables of sex, *APOE* genotype, and treatment), except for synaptic protein levels that by design were analyzed *via* Student’s *t*-test. We have plotted all data based on the results of statistical analysis; however, we have also included graphs with the data stratified by sex, *APOE* genotype, and treatment in Supplementary File 2.

### Body Weight and Food Intake of Female and Male *APOE3* and *APOE4* Mice Treated With LT-Krill Oil

We initially evaluated whether there were any changes in body weight or food consumption between mice treated with LT-krill oil, krill oil, or control diets. There was an effect of treatment on the percent change in body weight from initial treatment (4 months of age) to treatment endpoint (12 months of age). *Post hoc* analysis revealed that the treatment effect was driven by a slightly greater increase in percent body weight change (6%) for mice treated with krill oil compared with control diets; however, there were no differences between LT-krill oil treatment and control treatment (Supplementary Figure 1A). Treatment and *APOE* genotype independently modulated average food consumption (over 8 months); mice treated with krill oil and LT-krill oil mice consumed ∼10% more food than control diet, and *APOE3* mice consumed ∼3.3% more than *APOE4* mice (Supplementary Figure 1B). Overall, these data suggest no difference in general body weight, but slightly higher food consumption with LT-krill oil treatment.

### Plasma and Brain DHA and EPA Levels Were Enriched in *APOE3* and *APOE4* Mice Treated With LT-Krill Oil

We next evaluated the effect of LT-krill oil on the percentage of DHA and EPA (of total fatty acids) in the plasma and brain by GC/MS, as well as the isomer (LPC-DHA and LPC-EPA) composition by LC/MS.

#### DHA Levels

**FIGURE 1:**
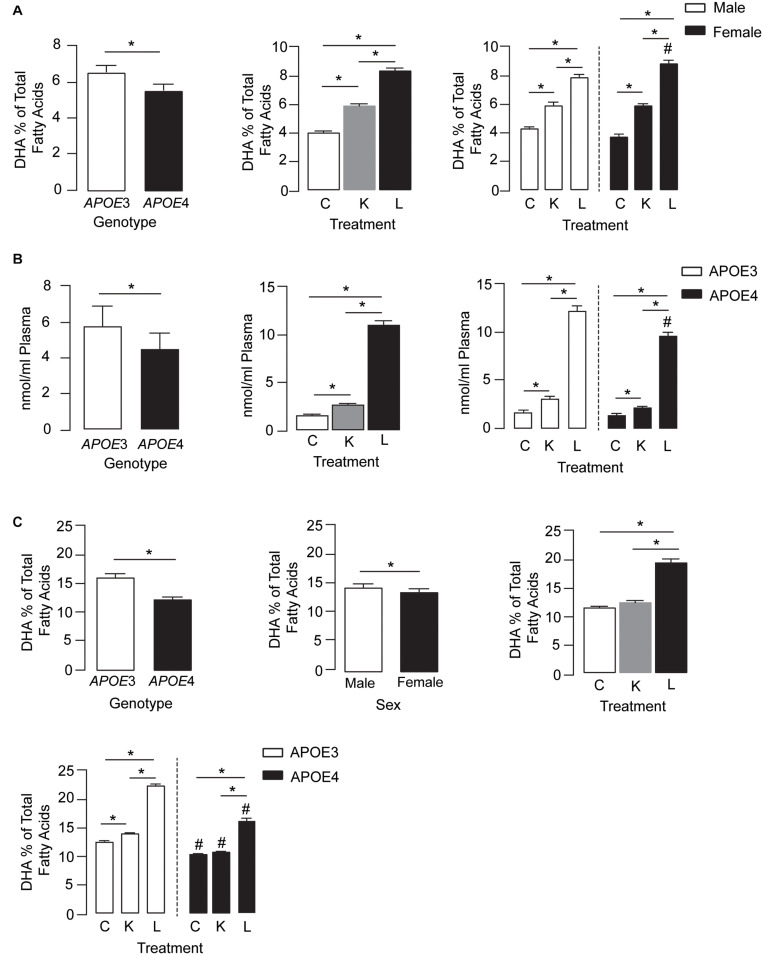
LT-krill oil treatment resulted in enriched plasma and brain levels of DHA in male and female *APOE3*-TR and *APOE4*-TR mice. **(A)** The DHA percentage of total fatty acids in the plasma was lower in *APOE4* mice than in *APOE3* mice [*F*_(2, 48)_ = 108.9, *p* < 0.0001] and differed between mice treated with LT-krill oil, krill oil, and control diets [*F*_(2, 48)_ = 669.7, *p* < 0.0001]. In addition, there was a treatment × sex interaction [*F*_(2, 48)_ = 21.58, *p* < 0.0001]. In both male and female mice, LT-krill oil treatment resulted in higher plasma levels of DHA than krill oil (males: *p* < 0.0001; females: *p* < 0.0001) or control (males: *p* < 0.0001; females: *p* < 0.0001). However, female mice treated with LT-krill oil had higher plasma DHA levels than male mice treated with LT-krill oil (*p* = 0.022). **(B)** Plasma concentrations of LPC-DHA (nmol/ml plasma) were lower in *APOE4* female mice than in *APOE3* female mice [*F*_(1, 30)_ = 29.56, *p* < 0.0001] and were altered by treatment [*F*_(1, 30)_ = 655.9, *p* < 0.0001] in the order of LT-krill oil > krill oil > control diets. Treatment × genotype interaction also impacted plasma concentrations of LPC-DHA [*F*_(1, 30)_ = 8.91, *p* = 0.0009]; the magnitude of the increase in LPC-DHA levels with LT-krill oil treatment was larger for *APOE3* than *APOE4* mice. **(C)** *APOE* [*F*_(2, 60)_ = 389.1, *p* < 0.0001], sex [*F*_(2, 60)_ = 16.63, *p* = 0.0001], treatment [*F*_(2, 60)_ = 655.6, *p* < 0.0001], treatment × *APOE* genotype [*F*(2, 60) = 36.32, *p* < 0.0001], and treatment × sex [*F*_(2, 60)_ = 4.27, *p* = 0.018] all impacted hippocampal DHA levels. For the independent effects, DHA levels were lower with *APOE4* compared with *APOE3*, in females compared with males, and followed the order LT-krill oil > krill oil = control. The treatment × *APOE* genotype interaction was likely driven by the fact that that krill oil alone enriched hippocampal DHA levels in *APOE3* but not in *APOE4* mice, and that for every treatment, DHA levels were higher in *APOE3*-TR mice. All data expressed as mean ± SEM. *p < 0.05 by three-way ANOVA and Tukey’s *post hoc* analysis. ^#^Different from APOE3 by Tukey’s *post hoc* analysis, *p* < 0.05. See Supplementary Table 1 for details on *n* sizes and statistical comparisons. C, control diet; K, krill oil diet; L, lipase-treated krill oil diet.

Plasma DHA levels were modulated by *APOE* genotype, treatment, and treatment by sex effects (Figure 1A, left, middle, and right graphs, respectively). For the *APOE* genotype, plasma DHA levels were ∼18% lower in *APOE4* mice than in *APOE3* mice across all treatment conditions. Importantly, DHA levels in the plasma followed the order LT-krill oil > krill oil > controls. Interestingly, compared with control diets, LT-krill oil treatment resulted in a larger increase in plasma DHA levels in female mice (2.3-fold) than in males (1.8-fold) (Figure 1A).

We also measured plasma LPC-DHA levels in a subset of female mice and found that levels were modulated by *APOE* genotype, treatment, and treatment by genotype effects (Figure 1B, left, middle, and right graphs, respectively). Consistent with total plasma DHA, the levels of LPC-DHA were ∼22% lower in *APOE4* mice than in *APOE3* mice and followed the order LT-krill oil > krill oil > controls. Additionally, LT-krill oil resulted in a slightly larger increase in plasma LPC-DHA levels in *APOE3* mice (7.1-fold) than in *APOE4* mice (6.6-fold) (Figure 1B). Thus, in general, plasma DHA levels and LPC-DHA levels are lower with *APOE4* and were increased after treatment with diets enriched in LPC-DHA.

We next determined whether the higher plasma DHA and LPC-DHA levels in mice treated with LT-krill oil diets translated to higher brain levels, and we focused on the hippocampus due its role in learning and memory. *APOE*, sex, treatment, treatment × *APOE* genotype, and treatment × sex all impacted hippocampal DHA levels (Figure 1C). For independent effects, DHA levels in the hippocampus were lower with *APOE4* compared with *APOE3*, in females compared with males, and followed LT-krill oil > krill oil = control. The treatment × *APOE* genotype interaction was likely driven by the fact that that krill oil alone enriched hippocampal DHA levels in *APOE3*-TR but not *APOE4-*TR mice by 12% and that for every treatment DHA levels were higher in *APOE3*-TR mice. For example, the magnitude of hippocampal DHA enrichment was greater in *APOE3*-TR (1.8-fold) than in *APOE4*-TR mice (1.5-fold) for LT-krill oil diets (Figure 1C), consistent with plasma levels of LPC-DHA (Figure 1B, right).

Collectively, our data support that DHA levels in the plasma and hippocampus are lower in *APOE4*-TR mice and that LT-krill oil can increase DHA and LPC-DHA levels in the plasma and hippocampus of *APOE3*- and *APOE4*-TR mice.

#### EPA Levels

**FIGURE 2:**
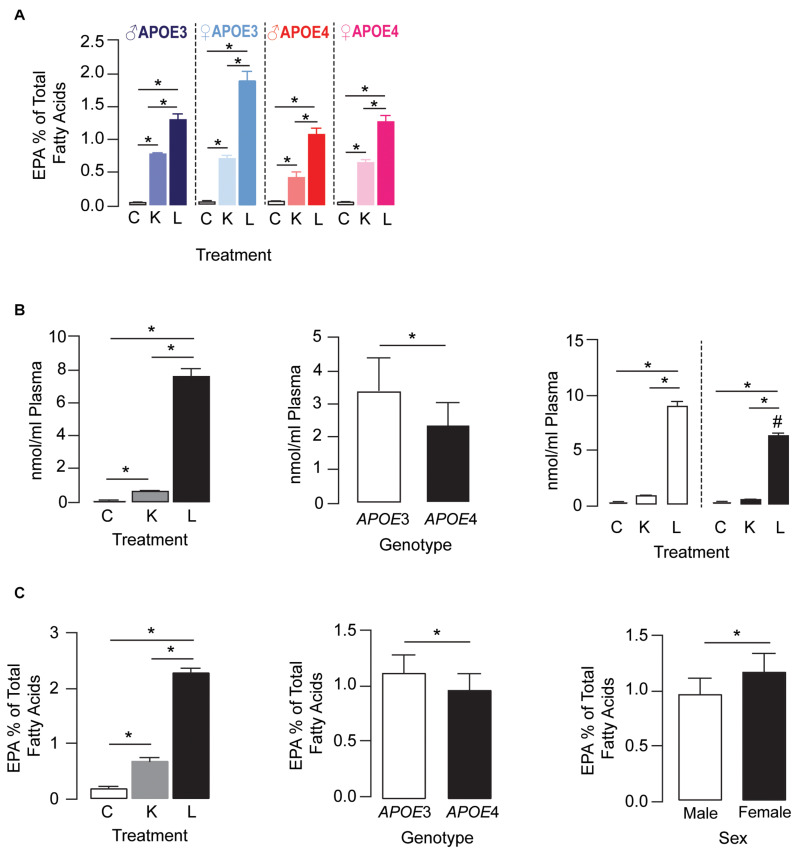
LT-krill oil treatment resulted in enriched plasma and brain levels of EPA in male and female *APOE3*-TR and *APOE4*-TR mice. **(A)** For plasma EPA levels, there was a treatment × *APOE* genotype × sex interaction [*F*_(2, 48)_ = 5.89, *p* = 0.0051]. Plasma EPA levels followed the order LT-krill oil (15–34-fold) > krill oil (5–13-fold) > control within each genotype for a particular sex. *APOE* genotype and sex did not alter EPA levels in control diet groups; however, they did change the extent when plasma EPA was enriched with krill oil and LT-krill oil diets with EPA levels higher with *APOE3* and female sex. **(B)** Plasma concentrations of LPC-EPA (nmol/ml plasma) were impacted by treatment [*F*_(2, 30)_ = 808.8, *p* < 0.0001], genotype [*F*_(1__, 30)_ = 35.86, *p* < 0.0001], and treatment × *APOE* genotype interaction [*F*_(2, 30)_ = 23.91, *p* < 0.0001] in female *APOE3* and *APOE4* mice. For both genotypes, LT-krill oil resulted in higher plasma LPC-EPA concentrations than krill oil (*p* < 0.0001) or control diets (*p* < 0.0001), and across all treatments, plasma LPC-EPA concentrations were lower in *APOE4* than in *APOE3* mice. The treatment × *APOE* genotype interaction was likely driven by a larger increase in plasma LPC-EPA concentrations with LT-krill oil treatment in *APOE3* female mice (40-fold) compared with *APOE4* female mice (36-fold). **(C)** Treatment, *APOE* genotype, and sex independently affected hippocampal EPA levels as the levels followed LT-krill oil > krill oil > control and were lower with *APOE4* and female sex. All data expressed as mean ± SEM. *p < 0.05 by three-way ANOVA and Tukey’s *post hoc* analysis. ^#^Different from APOE3 by Tukey’s *post hoc* analysis, *p* < 0.05. See Supplementary Table 1 for details on *n* sizes and statistical comparisons. C, control diet; K, krill oil diet; L, lipase-treated krill oil diet.

For plasma EPA levels, there was a treatment × *APOE* genotype × sex interaction effect. Further analysis revealed that plasma EPA levels followed the order LT-krill oil (15–34-fold) > krill oil (5–13-fold) > control within each genotype for a particular sex (Figure 2A). *APOE* genotype and sex did not alter EPA levels in control diet groups; however, they did change the extent when plasma EPA was enriched with LT-krill oil diets, with EPA levels higher in *APOE3* female mice. Plasma LPC-EPA concentrations were also altered by treatment, *APOE* genotype, and treatment × *APOE* genotype interaction in female *APOE3* and *APOE4* mice (Figure 2B). For both genotypes, LT-krill oil resulted in higher plasma LPC-EPA concentrations than krill oil or control diets, and across all treatments, plasma LPC-EPA concentrations were lower in *APOE4* than in *APOE3* female mice. The treatment × *APOE* genotype interaction was likely driven by a larger increase in plasma LPC-EPA concentrations with LT-krill oil treatment in *APOE3* female mice (40-fold) compared with *APOE4* female mice (36-fold). Additionally, treatment, *APOE* genotype, and sex independently affected hippocampal EPA as the levels followed LT-krill oil > krill oil > control, were lower with *APOE4*, and were higher in females (Figure 2C). Overall, our data support the concept that LT-krill oil supplementation can enrich plasma and brain EPA levels in mice that express *APOE3* and *APOE4*.

### Memory-Relevant Behavior Was Improved in *APOE4* Mice Treated With LT-Krill Oil

**FIGURE 3:**
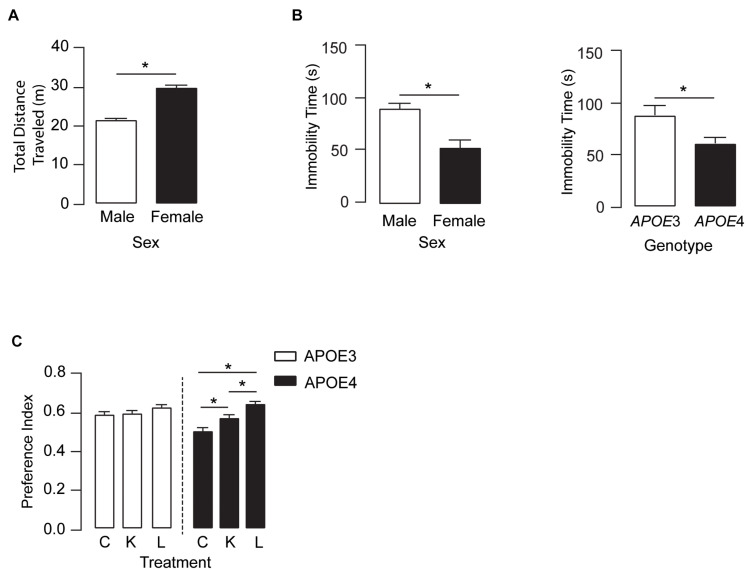
LT-krill oil treatment did not alter locomotion or anxiety-like behavior but resulted in improved memory-relevant behavior in male and female *APOE4* mice. **(A)** In the open filed test, there were no differences in total distance traveled between mice treated with LT-krill oil, krill oil, and control diets [*F*_(2, 216)_ = 1.19, *p* = 0.31]; however, female mice traveled greater total distances than male mice [*F*_(1, 216)_ = 76.94, *p* < 0.0001]. **(B)** There were also no differences in immobility time in the open field between mice treated with LT-krill oil, krill oil, and control diets [*F*_(2, 215)_ = 1.49, *p* = 0.23], but female mice spent less total time immobile than male mice [*F*_(1, 215)_ = 13.35, *p* = 0.0003] and *APOE4* mice spent less total time immobile than *APOE3* mice [*F*_(1, 215)_ = 6.33, *p* = 0.013]. **(C)** Treatment (LT-krill oil, krill oil, or control) altered performance on the novel object recognition task [*F*_(2, 184)_ = 11.02, *p* < 0.0001]; however, these effects were only observed in *APOE4* mice [*F*_(1, 184)_ = 3.80, *p* = 0.024]. In *APOE4* mice treated with LT-krill oil, performance was greater as compared with *APOE4* mice treated with control (*p* < 0.0001) and krill oil (*p* = 0.013). All data expressed as mean ± SEM. *p < 0.05 by three-way ANOVA and Tukey’s *post hoc* analysis. See Supplementary Table 1 for details on *n* sizes and statistical comparisons. C, control diet; K, krill oil diet; L, lipase-treated krill oil diet.

We next determined if the higher plasma and brain levels of DHA after LT-krill oil treatment were associated with improved behavior. In the open-field test, there were no treatment or *APOE* genotype effects in locomotor activity (Figure 3A). However, female mice traveled ∼38% farther than male mice and spent ∼70% less time immobile as compared with male mice, and *APOE4* mice across both sexes had a 36% lower immobility time as compared with *APOE3* mice. These data suggest that overall levels of anxiety-like behavior are higher in females and with *APOE4*, an effect that is not modulated by LPC-DHA treatment (Figure 3B). We next evaluated the effect of LT-krill oil treatment on memory-relevant behavior using the novel object recognition (NOR) test and identified a treatment and treatment × *APOE* genotype interaction. In *APOE4* mice, performance in NOR followed LT-krill oil > krill oil > control, whereas there was no difference in NOR performance among any of the diets in *APOE3* (Figure 3C). Indeed, performance was ∼13% higher with krill oil treatment and ∼28% higher with LT-krill oil treatment compared with controls in *APOE4* mice. These data support that LT-krill oil treatment, and to a lesser extent krill oil treatment, improves memory-relevant behavior in mice that express human *APOE4*.

### Hippocampal SV2A Levels Were Higher in Male and Female *APOE3* and *APOE4* Mice Treated With LT-Krill Oil

**FIGURE 4:**
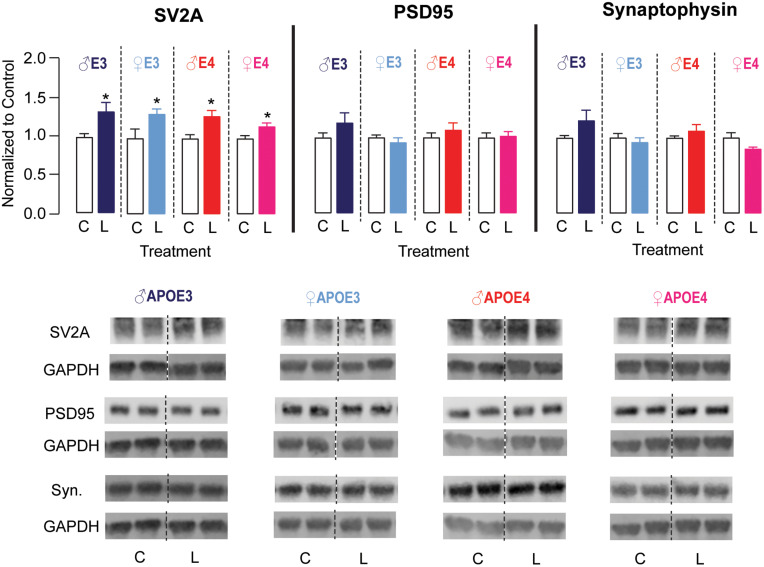
LT-krill oil treatment resulted in higher SV2A, but not PSD95 or synaptophysin, levels in the hippocampus of male and female *APOE3* and *APOE4* mice. Male and female *APOE3* and *APOE4* mice treated with LT-krill oil had higher hippocampal levels of SV2A [male *APOE3*: *t*(12) = 2.48, *p* = 0.029; female *APOE3*: *t*(10) = 2.23, *p* = 0.0503; male *APOE4*: *t*(12) = 3.01, *p* = 0.010; female *APOE4*: *t*(11) = 2.45, *p* = 0.032] compared with mice treated with control when assessed by Western blot analysis. Male and female *APOE3* and *APOE4* mice treated with LT-krill oil did not have altered hippocampal levels of PSD95 [male *APOE3*: *t*(12) = 1.29, *p* = 0.22; female *APOE3*: *t*(12) = 0.84, *p* = 0.42; male *APOE4*: *t*(12) = 0.85, *p* = 0.41; female *APOE4*: *t*(12) = 0.82, *p* = 0.82] or synaptophysin [male *APOE3*: *t*(12) = 1.59, *p* = 0.14; female *APOE3*: *t*(12) = 0.67, *p* = 0.52; male *APOE4*: *t*(12) = 1.01, *p* = 0.33; female *APOE4*: *t*(11) = 1.79, *p* = 0.10] compared with mice treated with control. PSD95 and synaptophysin were measured on the same Western blots, which is reflected in the GAPDH normalization control. All data expressed as mean ± SEM. *p < 0.05 by Student’s *t*-test. See Supplementary Table 1 for details on *n* sizes and statistical comparisons. C, control diet; L, lipase-treated krill oil diet.

The hippocampus is important for learning and memory, and DHA has been demonstrated to aid in maintaining neuronal function in the hippocampus (Cao et al., 2009; Hennebelle et al., 2014; Belkouch et al., 2016). Therefore, we evaluated whether LT-krill oil treatment altered synaptic protein levels in the hippocampus of male and female *APOE3* and *APOE4* mice by Western blot analysis (Figure 4). There were no changes in hippocampal levels of synaptophysin or PSD95, which are archetypical pre- and postsynaptic markers, respectively. We therefore measured the levels of SV2A, which is a synaptic vesicle protein and is emerging as a promising marker of synaptic dysfunction in AD patients (Chen et al., 2018; Mecca et al., 2020). We found that the levels of SV2A were ∼16–33% higher with LT-krill oil treatment compared with control treatment in male and female *APOE3* and *APOE4* mice. Therefore, in *APOE4* mice, the improved behavior with LT-krill oil correlated with higher SV2A levels in the hippocampus.

## Discussion

*APOE4* is associated with greater neuron and memory deficits in age-related neurodegenerative conditions. Therefore, identification of *APOE*-modulated factors that contribute to changes in brain function could aid in the development of preventive treatments for neurodegeneration. There is evidence of a connection between *APOE* and DHA during aging. DHA plays an important role in membrane homeostasis and intracellular signaling to aid in maintaining neuronal function throughout the lifespan (Cao et al., 2009; Cole et al., 2010; Bradbury, 2011; Weiser et al., 2016). As *APOE4* is associated with greater neuronal dysfunction during aging, DHA may be particularly important for neuroprotection. Indeed, it has been proposed that human *APOE4* carriers are more vulnerable to global dietary DHA deficiencies than *APOE3* and that diets enriched in DHA may be protective for *APOE4* carriers (Nock et al., 2017; Yassine et al., 2017a; Chappus-McCendie et al., 2019; Patrick, 2019). Human and mouse studies suggest that there is a more direct link between *APOE* and DHA metabolism. In humans, the whole-body half-life of DHA is lower and β-oxidation is higher with *APOE4* after acute treatment with radiolabeled DHA (Chouinard-Watkins et al., 2013), which has also been found in *APOE*-targeted replacement mice (Conway et al., 2014; Abdullah et al., 2017; Nock et al., 2017). Importantly, in *APOE*-TR mice, cortical DHA uptake is lower with *APOE4* after *in situ* cerebral perfusion (Vandal et al., 2014), and in our study, plasma and hippocampal DHA levels were lower with *APOE4*. Interestingly, there are reports in humans of higher brain DHA uptake with *APOE4*, which could be related to ongoing neuronal dysfunction that required higher DHA levels for reparative process (Yassine et al., 2017b). Through drawing these concepts together, it is possible that during aging, *APOE4* carriers have a greater requirement for DHA to maintain neuronal function; however, dietary DHA is metabolized in a way that does not result in sufficient brain enrichment. For example, with *APOE4*, there could be a lower supply of DHA to the neurons due to higher plasma clearance, lower transport across the blood–brain barrier, and altered trafficking in the brain due to lower lipidation of brain apoE (Nock et al., 2017; Yassine et al., 2017a; Chappus-McCendie et al., 2019; Patrick, 2019). Future studies could reveal the extent that *APOE*-modulated changes in DHA metabolism contributes to learning and memory dysfunction during aging and neurodegenerative disorders.

Evidence supporting a connection between *APOE* and DHA metabolism led to observational studies and clinical trials to test whether long-term DHA supplementation lowers dementia risk or symptoms (reviewed in Yassine et al., 2017a). Observational studies in healthy participants and Alzheimer’s disease patients have produced mixed results on the effect of DHA supplements on cognition and dementia risk, with no effects for any *APOE* genotype, beneficial effects only for *APOE4* carriers, and only for *APOE4* non-carriers all reported (Yassine et al., 2017a). In general, clinical trials evaluating the activity of DHA treatments in patients with Alzheimer’s disease or dementia have been negative. Specific for *APOE*, in the Alzheimer’s Disease Cooperative Study (ADCS), DHA failed to impact learning and memory in all patients; however, there was an indication of a beneficial effect in *APOE4* non-carriers. The lack of an effect of DHA in *APOE4* carriers may be related to altered DHA metabolism. Indeed, in the subgroup analysis of the ADCS-DHA trial, it was found that plasma and CSF levels of DHA were lower in *APOE4* carriers after 18 months of treatment (Tomaszewski et al., 2020). Therefore, higher amounts of DHA in supplements, or more frequent dosing, may be needed to effectively enrich DHA levels with *APOE4*. In mouse studies, long-term treatment of *APOE4*-TR mice with diets enriched with DHA or fish oil (both ∼0.7 g DHA/100 g diet) resulted in ∼9–20% increase in hippocampal levels of DHA and improvements in memory-relevant behavioral assays (Kariv-Inbal et al., 2012). In the aforementioned studies, total DHA levels in the diet were ∼8 times higher than in the current study, which supports that in addition to total levels, utilizing diets that contain LPC-DHA is another way to enrich brain DHA. In addition, in the context of neurodegenerative disorders, both higher or more frequent doses and LPC-DHA diets may be needed to enrich brain DHA levels. For example, in our study, the enrichment of brain DHA was generally higher with *APOE3* compared with *APOE4* with LT-krill oil, and pathological changes at the blood–brain barrier and in the brain may further impact both the metabolism/transport of DHA into the brain and the amount required to produce protective effects for neurons. Thus, although clinical trials with currently available DHA supplements may not have produced the beneficial outcomes predicted for *APOE4* carriers, our study along with others support that there may be some benefit to optimizing supplements to enrich brain DHA to provide protection for neurodegenerative disorders.

There are some limitations and unresolved questions raised by our current study, the first of which surrounds the mechanism of how the LT-krill oil diet improved memory and neuronal function. The most likely is that higher DHA levels (with LT-krill oil) modulated several pathways in the periphery and in the brain to result in improved neuron function in *APOE4*-TR mice, and further research could provide the mechanistic basis on how *APOE* and DHA interact to impact neuronal function and provide pharmacodynamic readouts in any subsequent trials. Related are additional critical questions on the ability of LT-krill oil to improve neuron function and behavior for other *APOE* genotypes and with more advanced neuronal dysfunction. Related is the possibility that *APOE4* carriers produce less LPC-DHA from TG-DHA in the liver, resulting in lower brain DHA levels, and LPC-DHA treatment can overcome this effect. Ultimately, addressing these questions is key in identifying the conditions where DHA could prevent neuronal dysfunction. Due to the design of the study, we are not able to distinguish the effects of EPA vs. DHA on neuronal markers and behavior in *APOE*4 mice. Although LT-krill oil results in relatively higher enrichment of brain DHA compared with EPA, EPA is thought to be beneficial for a range of peripheral and brain relevant functions and conditions (e.g., depression, cardiovascular disease). However, acute free (non-LPC) EPA treatment has been reported to impair learning and memory as well as hippocampal function in mice, which is prevented by co-treatment with DHA (Liu et al., 2020). Although these data draw into question the acute benefit of EPA, EPA as free acid is taken up through by diffusion into the brain and is mostly oxidized (Chen and Bazinet, 2015), whereas EPA in the LPC form is transported by a specific transporter (Mfsd2a) and may be beneficial. Future studies could compare the activity of LPC-EPA, LPC-DHA, and combined LPC-EPA/DHA in acute and chronic treatment studies *in vivo* to fully dissect their effects on neuron function and behavior. We also identified a small but significant change in body weight and food consumption, in that krill oil-treated mice had a greater increase in body weight and food consumption compared with the control diet and LT-krill oil-treated mice also consumed more food than mice on control diet. There are many potential explanations for this effect, ranging from changes in long-term locomotor activity and metabolism to a preference in taste or texture for one diet over another. Therefore, an unresolved question is the extent and impact that weight change/food consumption has on brain function when evaluated in assays beyond those used in this manuscript.

In conclusion, our data support that diets containing high levels of LPC-DHA can enrich brain DHA and improve memory-relevant behavior in mice that express *APOE4*. Therefore, long-term use of supplements that contain high LPC-DHA levels may on some level protect against age-related neurodegeneration.

## Data Availability Statement

The raw data supporting the conclusions of this article will be made available by the authors, without undue reservation.

## Ethics Statement

The animal study was reviewed and approved by the UIC Institutional Animal Care and Use Committee.

## Author Contributions

SBS, LMT, and PVS conceived and designed the experiments, performed the experiments, analyzed and interpreted the data, and wrote the manuscript. MZ, DS, PCRY, and FMM performed the experiments. All authors contributed to the article and approved the submitted version.

## Conflict of Interest

The authors declare that the research was conducted in the absence of any commercial or financial relationships that could be construed as a potential conflict of interest.
