# Supplementary material for: LPC-DHA/EPA-Enriched Diets Increase Brain DHA and Modulate Behavior in Mice That Express Human APOE4
Source: Front Neurosci. 2021 Jul 1;15:690410. doi: 10.3389/fnins.2021.690410 (PMC8282213; doi:10.3389/fnins.2021.690410)
Supplement: Supplementary file 1 [file Data_Sheet_1.PDF]

Supplementary Material

Supplementary Figures

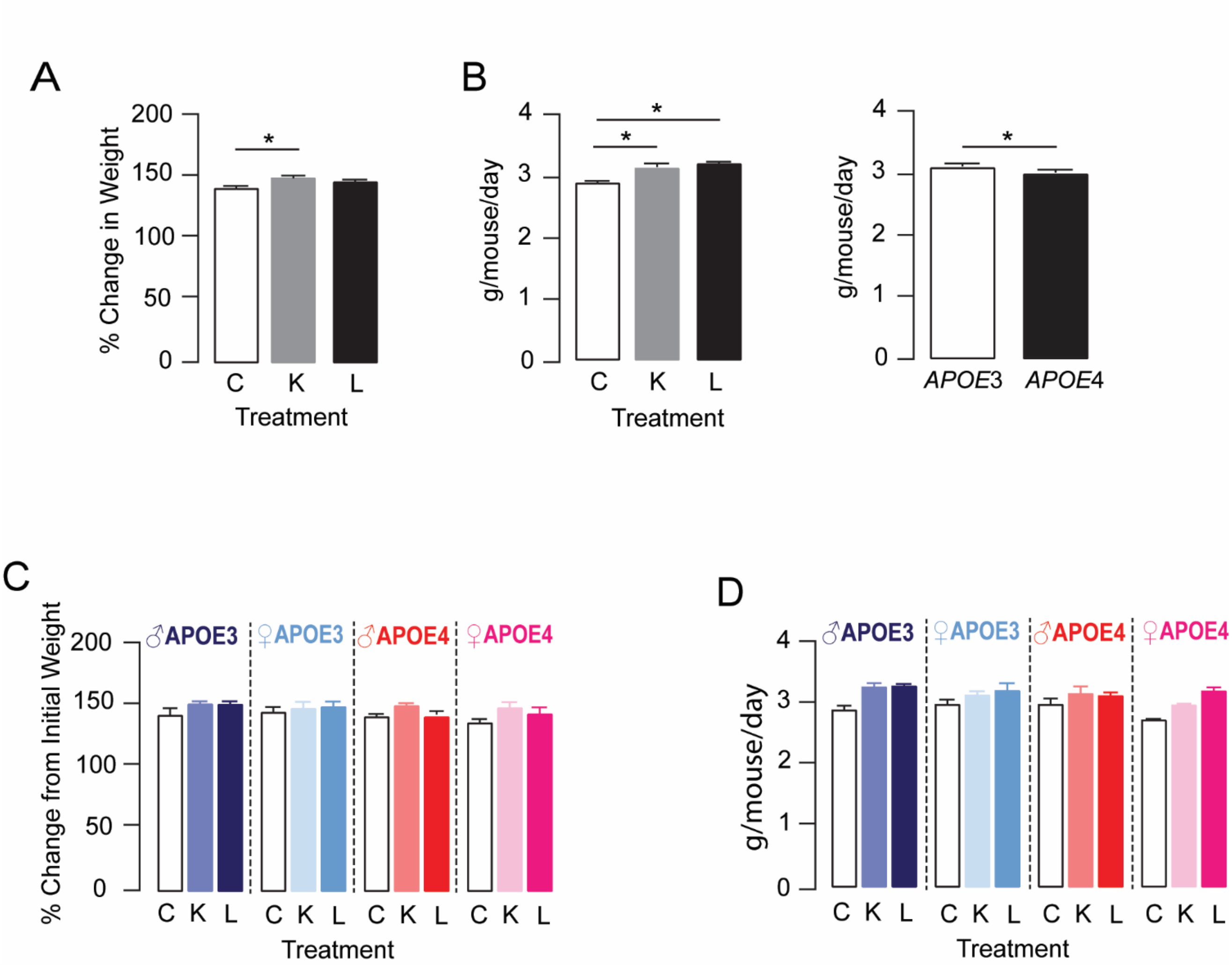

Supplementary Figure 1. Effect of LT-krill oil treatment on body weight and food intake in male and female *APOE3*-TR and *APOE4*-TR mice. (A) Treatment (LT-krill oil, krill oil, or control) altered the percent change in body weight from 4 months of age to 12 months of age ($F(2, 217)=4.33$, $p=0.014$). Mice treated with krill oil ($p=0.012$), but not LT-krill oil ($p=0.13$) had greater increases in body weight over the course of the study compared to controls. **(B)** Treatment altered food consumption ($F(2, 218)=17.16$, $p<0.0001$), as mice treated with krill oil ($p<0.0001$) and LT-krill oil ($p<0.0001$) consumed more food than those treated with control diet. Further, *APOE* genotype had an effect on food intake ($F(2, 218)=4.75$, $p=0.030$), with *APOE4* mice consuming less food compared to *APOE3* mice. **(C)** Percent change from initial weight and **(D)** food consumption stratified by sex, *APOE* genotype, and treatment. All data are expressed as the mean +/- SEM; *$p<0.05$ by three-way ANOVA

and Tukey's post-hoc analysis. See Supplementary Table 1 for details on *n* sizes and statistical comparisons. C=Control diet K=Krill oil diet L=Lipase-treated krill oil diet

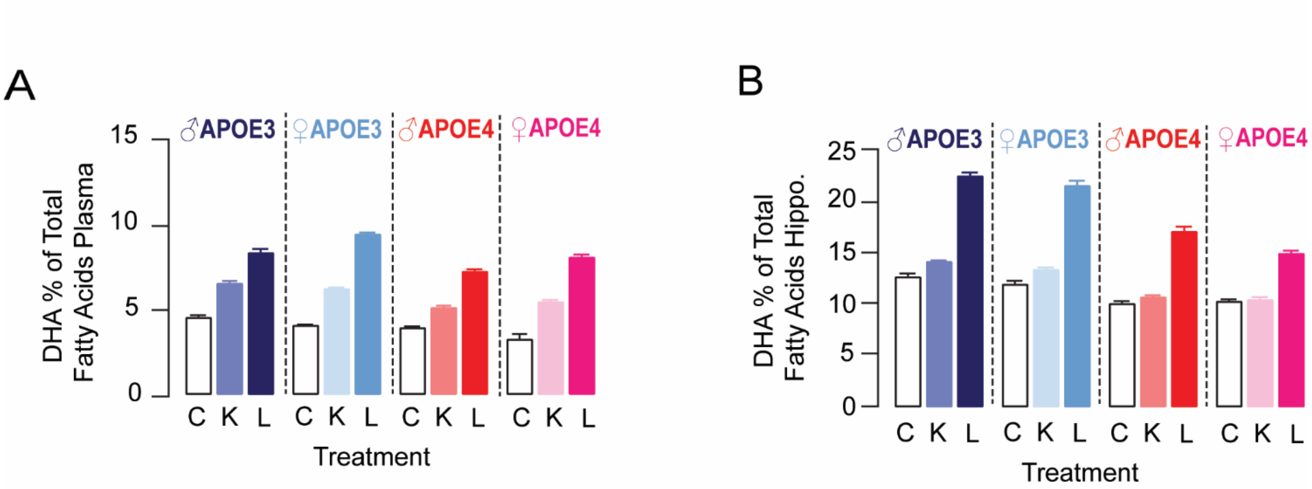

Supplementary Figure 2. Plasma and brain DHA levels by sex, *APOE* genotype and treatment. **(A)** Plasma DHA levels and **(B)** hippocampal DHA levels stratified by sex, *APOE* genotype, and treatment. All data are expressed as the mean +/- SEM. C=Control diet K=Krill oil diet L=Lipase-treated krill oil diet

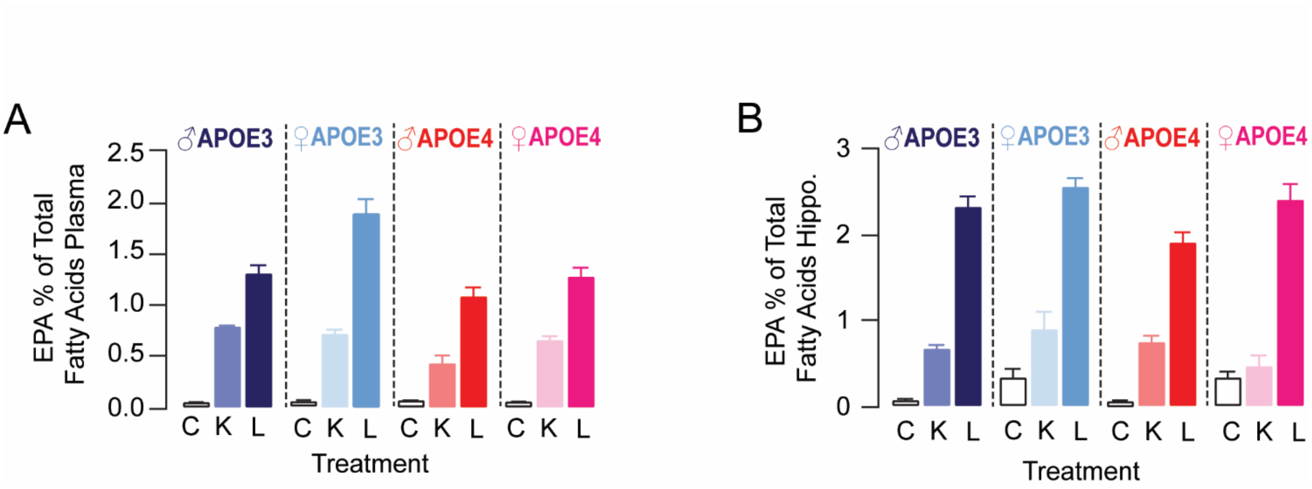

Supplementary Figure 3. Plasma and brain EPA levels by sex, *APOE* genotype and treatment. **(A)** Plasma EPA levels and **(B)** hippocampal EPA levels stratified by sex, *APOE* genotype, and treatment. All data are expressed as the mean +/- SEM. C=Control diet K=Krill oil diet L=Lipase-treated krill oil diet

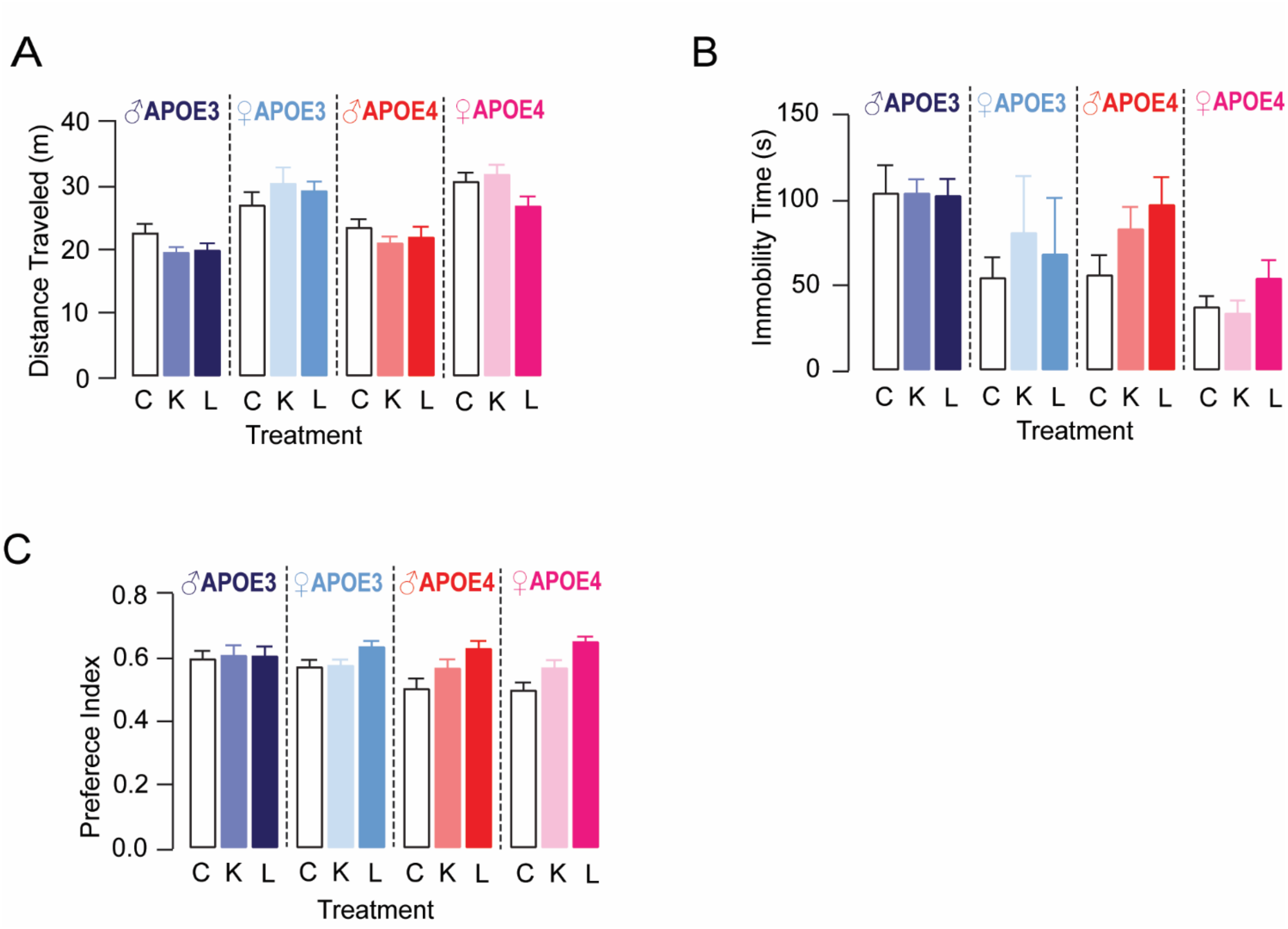

Supplementary Figure 4. Open field and novel object recognition readouts by sex, *APOE* genotype and treatment. **(A)** Total distance traveled in the open field test, **(B)** immobility time in the open field test, and **(C)** preference index on the novel object recognition test stratified by sex, *APOE* genotype and treatment. All data are expressed as the mean +/- SEM. C=Control diet K=Krill oil diet L=Lipase-treated krill oil diet
